# Investigation of geographic disparities of pre-diabetes and diabetes in Florida

**DOI:** 10.1186/s12889-020-09311-2

**Published:** 2020-08-12

**Authors:** Jennifer Lord, Shamarial Roberson, Agricola Odoi

**Affiliations:** 1 Department of Biomedical and Diagnostic Sciences, College of Veterinary Medicine, The University of Tennessee, Knoxville, TN USA; 2 Bureau of Chronic Disease Prevention, Division of Community Health Promotion, Florida Department of Health, Tallahassee, FL USA

**Keywords:** Pre-diabetes, Diabetes, Spatial epidemiology, Geographic disparities, Behavioral Risk Factor Surveillance System, Florida, Spatial scan statistics, SaTScan, Logistic regression

## Abstract

**Background:**

Diabetes is a leading cause of death and disability in the United States, and its precursor, pre-diabetes, is estimated to occur in one-third of American adults. Understanding the geographic disparities in the distribution of these conditions and identifying high-prevalence areas is critical to guiding control and prevention programs. Therefore, the objective of this study was to investigate clusters of pre-diabetes and diabetes risk in Florida and identify significant predictors of the conditions.

**Methods:**

Data from the 2013 Behavioral Risk Factor Surveillance System were obtained from the Florida Department of Health. Spatial scan statistics were used to identify and locate significant high-prevalence local clusters. The county prevalence proportions of pre-diabetes and diabetes and the identified significant clusters were displayed in maps. Logistic regression was used to identify significant predictors of the two conditions for individuals living within and outside high-prevalence clusters.

**Results:**

The study included a total of 34,186 respondents. The overall prevalence of pre-diabetes and diabetes were 8.2 and 11.5%, respectively. Three significant (*p* < 0.05) local, high-prevalence spatial clusters were detected for pre-diabetes, while five were detected for diabetes. The counties within the high-prevalence clusters had prevalence ratios ranging from 1.29 to 1.85. There were differences in the predictors of the conditions based on whether respondents lived within or outside high-prevalence clusters. Predictors of both pre-diabetes and diabetes regardless of region or place of residence were obesity/overweight, hypertension, and hypercholesterolemia. Income and physical activity level were significant predictors of diabetes but not pre-diabetes. Arthritis, sex, and marital status were significant predictors of diabetes only among residents of high-prevalence clusters, while educational attainment and smoking were significant predictors of diabetes only among residents of non-cluster counties.

**Conclusions:**

Geographic disparities of pre-diabetes and diabetes exist in Florida. Information from this study is useful for guiding resource allocation and targeting of intervention programs focusing on identified modifiable predictors of pre-diabetes and diabetes so as to reduce health disparities and improve the health of all Floridians.

## Background

Diabetes and pre-diabetes are defined by fasting plasma glucose (FPG) levels of 126 mg/dL or higher, and 100 mg/dL to < 126 mg/dL, respectively [1]. Persons with pre-diabetes have a significantly higher risk of developing diabetes in comparison to those with normal FPG levels. Without early intervention, 70% of pre-diabetes cases will progress to diabetes within 10 years [2, 3]. Complications of diabetes represent a significant public health burden and can be either: (a) microvascular complications such as neuropathy, nephropathy, and ocular damage; or (b) macrovascular complications such as cardiovascular disease [4–9].

In the United States, there are 30.3 million people with diabetes and 84.1 million with pre-diabetes [10]. Diabetes-related mortality is considered the seventh leading cause of death in the United States [11]. However, a recent study suggests that the contribution of diabetes to national mortality is severely underestimated, and actually approaches 12% among adults in the United States; this would move its rank to the third leading cause of death, following heart disease and malignant neoplasms [12, 13]. In the United States, diabetes and related complications are estimated to result in direct medical costs totaling $237 billion, and $90 billion of indirect costs due to reduced productivity from causes including disability, absenteeism, and early mortality [14].

As of 2016, Florida had 1.9 million people living with diabetes and 1.4 million with pre-diabetes. This was the second-highest (following Texas) number of people with pre-diabetes in any U.S. state [15]. The American Diabetes Association estimates annual diabetes costs in Florida to be $25 billion [16]. Moreover, the prevalence of pre-diabetes and diabetes in Florida has been consistently higher than the national average for years [15]. For instance, in 2016 the age-adjusted prevalence estimates of pre-diabetes and diabetes in Florida were 8.7 and 9.8%, respectively, compared to 7.4% (pre-diabetes) and 9.2% (diabetes) nationwide [15].

In 2011, the Centers for Disease Control and Prevention (CDC) identified the “diabetes belt”, a geographic region with a higher prevalence of diabetes (11.7%) than the national average (8.5%) [17]. The diabetes belt is comprised of a cluster of 644 counties in 15 states, primarily in the Southeastern U.S.: Alabama, Arkansas, Florida, Georgia, Kentucky, Louisiana, Mississippi, North Carolina, Ohio, Pennsylvania, South Carolina, Tennessee, Texas, Virginia, and West Virginia [17]. In these counties, 30% of the excess risk of diabetes is associated with modifiable, “lifestyle” risk factors and 37% with non-modifiable, hereditary risk factors [17]. Understanding the geographic distribution of diabetes prevalence is important in guiding resource allocation and intervention programs to combat the problems as it would help in identifying local areas at high risk that would require targeted intervention to reduce disparities. Unfortunately, very few studies have used rigorous spatial epidemiologic/statistical investigations (beyond basic mapping) that would generate useful information to guide targeted health programs. Conducting such studies at sub-state levels will be critically important in providing useful information to help curb this epidemic. Therefore, the objectives of this study were to investigate geographic disparities of pre-diabetes and diabetes prevalence and identify predictors of the two conditions in Florida. These approaches would be critical in helping to meet one of the goals of the Healthy People 2020 which is to reduce health disparities and improve health of the whole population.

## Methodology

### Study area

**Fig. 1:**
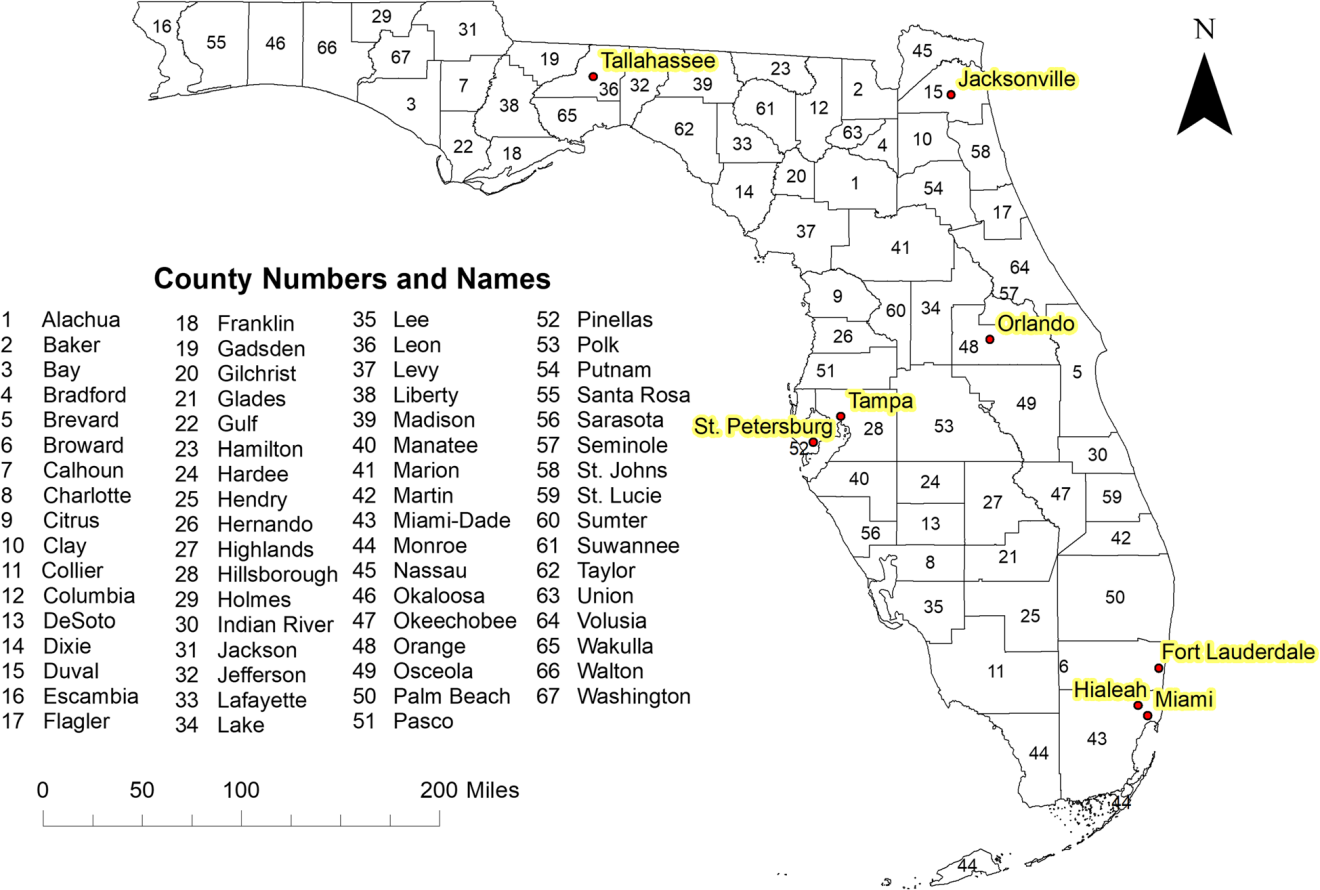
Geographic distribution of counties and major cities in Florida, USA. The map was created by the authors of the study using ArcGIS software [18]. The geographic boundary file was obtained from the United States Census Bureau TIGER Geodatabase [19]. These files are publicly available, and permission is not required to reproduce the image

This study was performed in the state of Florida, which consists of 67 counties (Fig. 1), many of which are a part of the diabetes belt and have high prevalence proportions of pre-diabetes and diabetes [17]. As of the 2010 population census, Florida had approximately 18.8 million residents, 75% of whom were white, 16% were black and 9% were of other races [20]. Forty nine percent of Florida residents are male while the other 51% are female [20]. Florida has an even age distribution among adults, with 24% of the population comprising young adults 18–34 years old, 26% are 35–49 years old, 25% are 50–64 years old, and 22% are seniors (≥65 years of age). The state is made up of both urban and rural areas. Miami-Dade County, the southern-most county on the east coast, is the most urban and the most populated, with 2.5 million residents [20]. Liberty County, located to the west of Tallahassee, is the most rural and least populated with a population of 8365 [20]. County land area in Florida ranges from 243.6 mile^2^ (Union County) to 1998 mile^2^ (Collier County) [19].

### Pre-diabetes and diabetes data

Data from the Behavioral Risk Factor Surveillance System (BRFSS) for the period from January to December 2013 were obtained from the Florida Department of Health. Diabetes status was based on self-reports from individual respondents who reported being told by a doctor that they had pre-diabetes or diabetes, not related to pregnancy. No distinction was made between type 1 and 2 diabetes. Data obtained for each respondent included county of residence, age, gender, race, marital status, body mass index (BMI), physical activity level, smoking status, fruit intake, vegetable consumption, hypertension, high cholesterol, arthritis, educational attainment, income level and health insurance. Pre-diabetes and diabetes data were aggregated to the county level and exported to ArcGIS [18].

### Descriptive analyses

Descriptive analyses were performed in SAS 9.4 [21]. Shapiro-Wilk test was used to assess for normality of distribution of continuous variables. When continuous variables were non-normally distributed, median and interquartile ranges were used for descriptive statistics. Since the data used in this study is from a complex survey, all frequency calculations were done using the survey frequency procedure for complex survey data, PROC SURVEYFREQ, in SAS. This allowed adjustment for strata, clusters and sampling weights.

### Cluster identification and investigation

Kulldorff’s spatial scan statistics was used to detect high-prevalence spatial clusters of pre-diabetes and diabetes and identify their locations using SaTScan v9.4.2 [22]. Using a Poisson model, the maximum spatial cluster size was set to 13% of the total study population at risk, based on the most populated county, Miami-Dade, and clusters were identified based on a likelihood ratio test. For statistical inference, 999 Monte Carlo replications were performed for all analyses, and the null hypothesis of complete spatial randomness was rejected at a *p*-value of ≤0.05. A significant high-prevalence cluster was interpreted as having higher prevalence of pre-diabetes or diabetes within the circular window relative to outside the window. Only clusters with a prevalence ratio ≥ 1.2 were reported to avoid reporting very low-prevalence clusters.

### Logistic regression

All regression modeling was performed in SAS 9.4 [21] using the survey logistic procedure, PROC SURVEYLOGISTIC, to adjust for the complex sampling strategy used for data collection. The outcomes of interest for regression modeling were binary, reflecting whether a survey respondent reported having pre-diabetes/diabetes or not. Four logistic models were built: (a) pre-diabetes model for respondents living within high-prevalence clusters, (b) pre-diabetes model for respondents living outside high-prevalence clusters, (c) diabetes model for respondents living within high-prevalence clusters, and (d) diabetes model for respondents living outside high-prevalence clusters. Each of the logistic models were built using manual backwards elimination approach using a critical *p*-value of ≤0.05. Non-significant variables were considered potential confounders if their removal from the model resulted in > 20% change in the coefficients of any of the remaining variables in the model and would be considered for retention in the final model. Biologically meaningful two-way interaction terms of variables included in the final main effects model were assessed for significance with the goal of retaining significant ones in the models. However, none were significant and hence no interaction terms were retained in the final models.

### Cartographic displays

All geographic information system (GIS) manipulations and cartographic displays were performed in ArcGIS [18]. The geographic boundary file used in this study was downloaded from the United States Census Bureau TIGER Geodatabase [19]. Pre-diabetes and diabetes prevalence were displayed in choropleth maps. Predictors of pre-diabetes and diabetes that were found to be significant in the logistic regression models were also displayed in choropleth maps. Critical intervals in the choropleth maps were determined using Jenk’s optimization classification scheme. Significant spatial clusters of high pre-diabetes and diabetes prevalence were also displayed using ArcGIS [18].

## Results

### Descriptive analyses

A total of 34,186 respondents participated in the BRFSS survey and were included in the study. The prevalence of diabetes was 11.5% while that of pre-diabetes was 8.2% (Table 1). Respondents ranged in age from 18 to 99 years, with a median of 61 and an interquartile range of 48 to 72. The prevalence of diabetes and pre-diabetes among seniors (≥65 years old) was 24.0 and 12.7%, respectively.

**Table 1:** Demographic, health, and lifestyle characteristics of adults in Florida, 2013

Characteristic	Unweighted Frequency	Weighted frequency	Weighted % (95% Confidence Interval)
**Diabetes status**	*n* = 34,108		
Diabetes	5189	1,736,908	11.45 (10.75, 12.20)
No diabetes	28,919	13,812,561	88.45 (87.80, 89.26)
**Pre-diabetes status**	*n* = 28,239		
Pre-diabetes	2983	1,052,085	8.23 (7.61, 8.88)
No pre-diabetes	25,256	11,929,511	91.77 (91.11, 92.40)
**BMI (kg/m**^**2**^**)**	*n* = 32,552		
Underweight (< 18.5)	720	335,135	2.27 (1.95, 2.64)
Normal (18.5–24.9)	10,815	5,162,640	34.96 (33.79, 36.15)
Overweight (25–29.9)	11,597	5,674,964	36.40 (35.20, 37.61)
Obese (≥ 30)	9420	3,895,148	26.38 (25.33, 27.44)
**Hypertension**	*n* = 34,074		
Yes	15,684	5,374,943	34.59 (33.50, 35.69)
No	18,390	10,165,863	65.41 (64.31, 66.50)
**Hypercholesterolemia**	*n* = 30,216		
Yes	14,445	5,087,114	40.33 (39.12, 41.56)
No	15,771	7,525,146	59.67 (58.44, 60.88)
**Arthriti**s	*n* = 33,897		
Yes	13,242	4,021,502	26.02 (25.11, 26.94)
No	20,655	11,436,638	73.98 (73.06, 74.89)
**Income level**	*n* = 29,171		
< $15,000	4222	1,953,171	14.40 (13.44, 15.43)
$15,000 - < $25,000	6390	2,765,754	20.40 (19.35, 21.48)
$25,000 - < $35,000	3798	1,686,773	12.44 (11.6, 13.33)
$35,000 - < $50,000	4415	1,994,956	14.71 (13.85, 15.62)
> $50,000	10,346	5,158,624	38.04 (36.83, 39.27)
**Health care coverage**	*n* = 34,003		
Yes	29,145	11,943,771	77.14 (76.01, 78.22)
No	4858	3,540,245	22.86 (21.78, 23.99)
**Race**	*n* = 34,186		
White non-Hispanic	27,368	9,291,500	59.61 (58.40, 60.80)
Black non-Hispanic	2947	2,170,793	13.93 (13.01, 14.89)
Other race non-Hispanic	1353	671,077	4.31 (3.87, 4.79)
Hispanic	2518	3,454,252	22.16 (20.96, 23.41)
**Age, years**	*n* = 34,186		
18–24	1377	1,798,079	11.54 (10.64, 12.50)
25–34	2518	2,412,500	15.48 (14.55, 16.46)
35–44	3196	2,397,353	15.38 (14.48, 16.33)
45–54	5149	2,741,414	17.59 (16.68, 18.53)
55–64	7331	2,573,989	16.51 (15.70, 17.36)
65 or older	14,615	3,664,287	23.51 (22.74, 24.29)
**Sex**	*n* = 34,186		
Male	13,340	7,538,722	48.36 (47.16, 49.57)
Female	20,846	8,048,900	51.64 (50.43, 52.84)
**Physical activity**	*n* = 28,197		
Highly Active (≥300 min of moderately intense or vigorous equiv./week)	9688	3,730,259	31.38 (30.18, 32.60)
Active (150-300 min of moderately intense or vigorous equiv./week)	4152	1,868,360	15.72 (14.75, 16.73)
Insufficiently Active (1-149 min of moderately intense exercise/week)	4245	1,968,192	16.55 (15.57, 17.59)
Inactive	10,112	4,322,146	36.35 (35.03, 37.70)
**Education**	*n* = 34,014		
< High school	3380	2,322,729	14.96 (13.91, 16.08)
High school	10,630	4,676,820	30.15 (29.03, 31.29)
Some college	9853	4,835,448	31.18 (30.11, 32.27)
College	10,151	3,677,142	23.71 (22.85, 24.59)
**Marital status**	*n* = 33,917		
Married	16,832	7,736,876	50.05 (48.86, 51.25)
Never married	4686	4,070,050	26.33 (25.18, 27.48)
Separated/divorced/ widowed	12,399	3,650,783	23.62 (22.65, 24.58)
**Consume vegetable(s)**	*n* = 30,315		
< 1 per day	5824	2,920,634	20.83 (19.80, 21.90)
> 1 per day	24,491	11,098,769	79.17 (78.10, 80.20)
**Consume fruit(s)**	*n* = 30,978		
< 1 per day	11,588	5,392,612	37.95 (36.73, 39.17)
> 1 per day	19,390	8,818,683	62.05 (60.83, 63.27)
**Smoked** **>** **100 cigarettes**	*n* = 33,078		
Yes	16,679	6,744,411	45.07 (43.88, 46.26)
No	16,399	8,219,768	54.93 (53.74, 56.12)

**Fig. 2:**
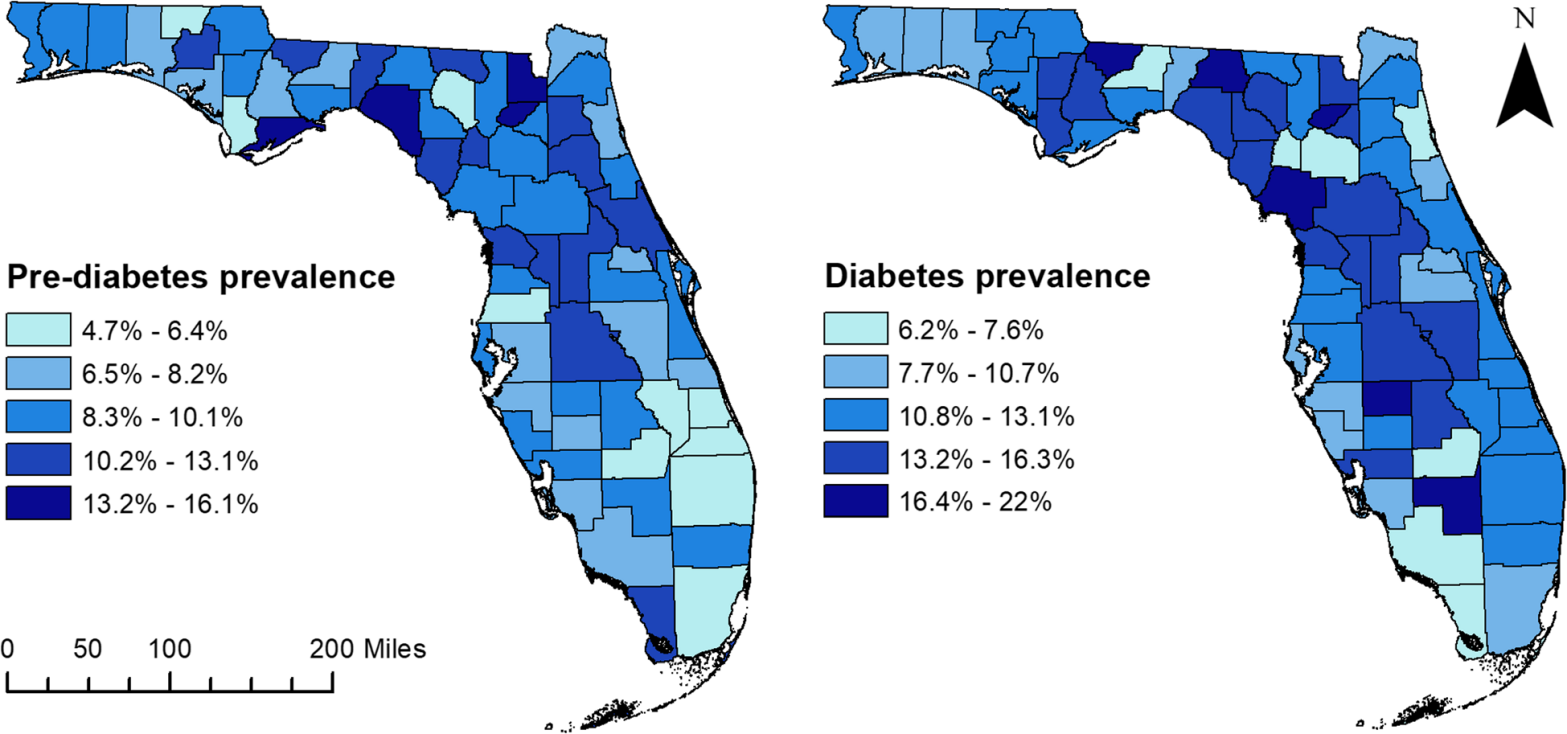
County-level prevalence of pre-diabetes and diabetes among adults in Florida, 2013. The map was created by the authors of the study using ArcGIS software [18]. The geographic boundary file was obtained from the United States Census Bureau TIGER Geodatabase [19]. These files are publicly available, and permission is not required to reproduce the image

County prevalence proportions of pre-diabetes and diabetes are displayed in Fig. 2. Pre-diabetes prevalence ranged from 4.7 to 16.1%, with a mean of 9.1%. Counties within the eastern panhandle tended to have higher prevalence proportions of pre-diabetes than counties in the western panhandle. Franklin and Taylor Counties, two rural counties, had the highest prevalence proportions of pre-diabetes in the panhandle (Figs. 1 and 2). In addition, Baker and Union Counties in northeast Florida, near the Jacksonville metropolitan area, also had high pre-diabetes prevalence (Figs. 1 and 2). There was also a swath of counties with high prevalence proportions extending from northern to central Florida, in a relatively metropolitan part of the state. Low prevalence proportions of pre-diabetes occurred primarily in urban metro areas such as Tampa (Hillsborough County) and Orlando (Orange County), as well as Miami (Broward and Miami-Dade Counties) and many of the southern coastal counties (Figs. 1 and 2).

Diabetes prevalence ranged from 6.2 to 21.9%, with a mean of 12.3% (Fig. 2). Many counties with high pre-diabetes prevalence proportions also had high diabetes prevalence proportions; however, the conditions did exhibit slightly different spatial patterns. Four major regions with relatively high diabetes prevalence could be discerned. In the panhandle, this included counties to the west of Tallahassee (Calhoun, Gadsen, Gulf, and Liberty Counties), as well as a swath of rural counties to the east of Tallahassee (Dixie, Lafayette, Levy, Madison, Suwanee and Taylor Counties) (Figs. 1 and 2). High prevalence proportions of diabetes also occurred in central Florida and extended to counties located inland in the south-central part of the state (Hardee, Hendry, Highlands, Osceola, and Polk Counties) (Figs. 1 and 2). Counties with relatively low diabetes prevalence were located within large metropolitan areas such as Jacksonville (Duval and St. Johns County), Miami (Broward and Miami-Dade Counties), Orlando (Orange County), Gainesville (Alachua and Gilchrist Counties), and Tallahassee (Leon County), as well as along the southern Gulf coast (Figs. 1 and 2).

### Clusters of pre-diabetes and diabetes

**Fig. 3:**
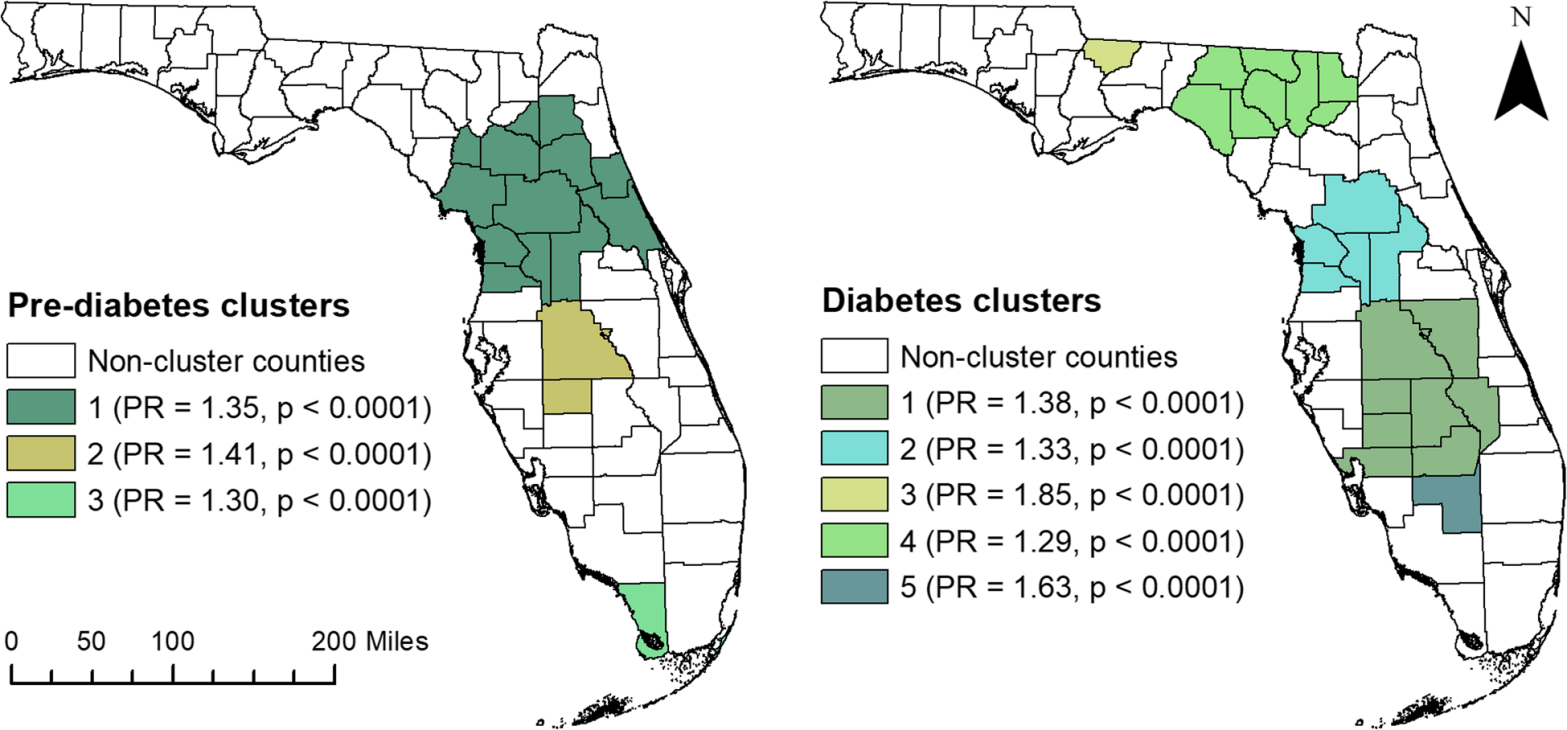
High-prevalence spatial clusters of pre-diabetes and diabetes at the county level among adults in Florida, 2013. The map was created by the authors of the study using SaTScan and ArcGIS software [18, 22]. The geographic boundary file was obtained from the United States Census Bureau TIGER Geodatabase [19]. These files are publicly available, and permission is not required to reproduce the image

Based on the results of spatial scan statistics, three significant (*p* < 0.001) pre-diabetes high-prevalence spatial clusters were identified (Table 2 & Fig. 3). The primary pre-diabetes cluster was composed of 13 counties in northern and central Florida to the east of the panhandle. The prevalence ratio (PR) of this cluster was 1.35, implying that the prevalence of pre-diabetes within the cluster was 35% higher than the prevalence in counties outside of the cluster. The prevalence proportion of pre-diabetes in the cluster area was 10.5% (Table 2). Two secondary pre-diabetes clusters were identified. Cluster 2 (PR = 1.41, *p* < 0.001) included Polk and Hardee counties and was directly adjacent to the south of the primary cluster (i.e. Cluster 1) (Figs. 1 and 3). Cluster 3 (PR = 1.30, *p* < 0.001) was composed of a single county, Monroe County, located at the southernmost tip of the state (Figs. 1 and 3). Detailed information on the characteristics of adults living within and outside high-prevalence pre-diabetes clusters are shown in Additional file 1.

**Table 2:** Purely spatial significant clusters of pre-diabetes and diabetes among adults in Florida, 2013

Cluster	Population	Observed Number of Cases	Expected Number of Cases	Prevalence (%)	Prevalence Ratio (PR)	***p***-value
Pre-diabetes Cluster 1	1,562,013	164,158	126,592.1	10.5	1.35	< 0.001
Pre-diabetes Cluster 2	401,956	45,335	32,576.2	11.3	1.41	< 0.001
Pre-diabetes Cluster 3	56,099	5885	4546.5	10.5	1.30	< 0.001
Diabetes Cluster 1	1,003,797	150,890	112,126.4	15.0	1.38	< 0.001
Diabetes Cluster 2	884,728	129,279	98,826.15	14.6	1.33	< 0.001
Diabetes Cluster 3	37,347	7704	4171.8	20.6	1.85	< 0.001
Diabetes Cluster 4	170,874	24,458	19,087.0	14.3	1.29	< 0.001
Diabetes Cluster 5	35,671	6490	3984.5	18.2	1.63	< 0.001

Five significant high-prevalence spatial clusters of diabetes were identified (Table 2 and Fig. 3). The primary diabetes cluster (Cluster 1) consisted of eight primarily rural counties located in south-central Florida, and was surrounded by the state’s largest metropolitan areas: Orlando to the north, Tampa to the west, and Miami to the southeast (Figs. 1 and 3). The prevalence of diabetes within this cluster was 38% higher (PR = 1.38; *p* < 0.001) than the prevalence of diabetes in counties outside the cluster. The prevalence proportion of diabetes within the primary cluster was 15% (Table 2). A secondary high-prevalence spatial cluster, Cluster 5 (PR = 1.63, *p* < 0.001), consisting of a single county (Hendry County) was immediately adjacent to the primary diabetes cluster (Figs. 1 and 3). Two high-prevalence diabetes clusters were also identified in the Florida panhandle. Cluster 3 (PR = 1.85, *p* < 0.001) consisted of a single county, Gadsden County, located to the west of Tallahassee in the central panhandle (Figs. 1 and 3). Cluster 4, located in the eastern panhandle, was composed of eight counties in the most rural region of the state (Figs. 1 and 3). This cluster had 29% higher prevalence (PR = 1.29, *p* < 0.001) of diabetes than counties outside the cluster. The cluster had an estimated diabetes prevalence of 14.3% (Table 2 & Fig. 3). Detailed information on the characteristics of adults living within and outside high-prevalence diabetes clusters are shown in Additional file 2.

### Logistic regression results

Tables 3 and 4 show the results of the final multivariable logistic models. Within both cluster and non-cluster counties, pre-diabetes status was significantly associated with BMI, hypertension, and hypercholesterolemia. Although the strength of association between pre-diabetes and obesity seemed to be greater among residents of non-cluster counties (OR = 3.89, 95% CI: 2.95, 5.13) in comparison to those living within cluster counties (OR = 2.67, 95% CI: 1.83, 3.90), the ORs were not significantly different since their confidence intervals overlap. However, being overweight was significantly associated with pre-diabetes among those living outside of high-prevalence clusters (OR = 2.12, *p* < 0.001), but not among residents of cluster counties (OR = 1.27; *p* = 0.291). On the other hand, although hypertension (OR = 2.37, 95% CI: 1.70, 3.30) and hypercholesterolemia (OR = 2.29, 95% CI: 1.62, 3.25) seemed to have stronger associations with pre-diabetes within cluster counties in comparison to the rest of the state (OR_hypertension_ = 1.80, 95% CI: 1.41, 2.29; OR_hypercholesterolemia_ = 1.93, 95% CI: 1.52, 2.46), again these were not significantly different as evidenced by the overlapping confidence intervals of the ORs.

**Table 3:** Predictors of pre-diabetes among adults residing within and outside high-prevalence clusters in Florida, 2013

Predictor	Model for Cluster Counties		Model for Non-cluster Counties	
	OR^**a**^ (95% CI^**b**^)	***p***-value	OR^**a**^ (95% CI^**b**^)	***p***-value
**BMI (kg/m**^**2**^**)**				
Obese (≥30)	2.67 (1.83, 3.90)	< 0.001	3.89 (2.95, 5.13)	< 0.001
Overweight (25–29.9)	1.27 (0.82, 1.97)	0.291	2.12 (1.60, 2.82)	< 0.001
Underweight (< 18.5)	1.75 (0.56, 5.46)	0.332	0.91 (0.35, 2.36)	0.839
Normal (18.5–24.9)	Referent	–	–	–
**Hypertension**				
Yes	2.37 (1.70, 3.30)	< 0.001	1.80 (1.41, 2.29)	< 0.001
No	Referent	–	–	–
**Hypercholesterolemia**				
Yes	2.29 (1.62, 3.25)	< 0.001	1.93 (1.52, 2.46)	< 0.001
No	Referent	–	–	–
**Race**				
Black, non-Hispanic	2.40 (1.19, 4.87)	0.015		
Hispanic	0.47 (0.28, 1.17)	0.127	–	–
Other race, non-Hispanic	1.76 (0.75, 4.14)	0.197		
White, non-Hispanic	Referent	–		
**Age**				
≥ 65 years	–	–	1.67 (1.28, 2.18)	0.001
55–64 years			1.43 (1.06, 1.94)	0.020
18–54 years			Referent	–
**Smoking status**				
≥ 100 lifetime cigarettes	1.49 (1.06, 2.08)	0.021	–	–
< 100 lifetime cigarettes	Referent	–		

^a^Odds ratio

^b^Confidence interval

**Table 4:** Predictors of diabetes among adults residing within and outside high-prevalence clusters in Florida, 2013

Predictor	Model for Cluster Counties		Model for Non-cluster Counties	
	OR^**a**^ (95% CI^**b**^)	***p-***value	OR^**a**^ (95% CI^**b**^)	***p***-value
**BMI (kg/m**^**2**^**)**				
Obese (≥30)	2.99 (2.04, 4.40)	< 0.001	3.68 (2.84, 4.76)	< 0.001
Overweight (25–29.9)	1.62 (1.09, 2.43)	0.019	2.06 (1.56, 2.73)	< 0.001
Underweight (< 18.5)	1.47 (0.54, 3.99)	0.445	0.40 (0.18, 0.88)	0.024
Normal (18.5–24.9)	Referent	–	Referent	–
**Hypertension**				
Yes	3.98 (2.98, 5.33)	< 0.001	2.44 (1.85, 3.23)	< 0.001
No	Referent	–	Referent	–
**Hypercholesterolemia**				
Yes	1.67 (1.27, 2.19)	< 0.001	1.86 (1.45, 2.39)	< 0.001
No	Referent	–	Referent	–
**Arthritis**				
Yes	1.48 (1.14, 1.93)	0.004	–	–
No	Referent	–		
**Income**				
< $15,000	3.34 (2.11, 5.27)	< 0.001	1.63 (1.10, 2.42)	0.015
$15,000 - < $25,000	1.65 (1.16, 2.37)	0.006	1.51 (1.13, 2.03)	0.006
$25,000 - < $35,000	1.84 (1.22, 2.78)	0.004	1.36 (0.96, 1.92)	0.085
$35,000 - < $50,000	1.18 (0.78, 1.79)	0.442	1.10 (0.81, 1.50)	0.532
≥ $50,000	Referent	–	Referent	–
**Age**				
≥ 65 years	2.20 (1.53, 3.16)	< 0.001	3.14 (2.32, 4.24)	< 0.001
55–64 years	1.79 (1.20, 2.68)	0.004	2.15 (1.55, 2.98)	< 0.001
18–54 years	Referent	–	Referent	–
**Sex**				
Male	1.68 (1.30, 2.19)	< 0.001	–	–
Female	Referent	–		
**Physical activity level**				
Inactive	1.47 (1.09, 1.98)	0.013	1.61 (1.24, 2.08)	< 0.001
Insufficiently active (1-149 min of moderately intense exercise/week)	1.64 (1.06, 2.53)	0.027	1.13 (0.83, 1.55)	0.438
Active (150-300 min of moderately intense or vigorous equiv./week)	1.46 (0.94, 2.28)	0.091	1.32 (0.95, 1.84)	0.102
Highly active (≥300 min of moderately intense or vigorous equiv./week)	Referent	–	Referent	–
**Educational attainment**	–	–		
Less than high school			1.76 (1.13, 2.74)	0.013
High school graduate			0.98 (0.75, 1.28)	0.883
Some college			0.97 (0.75, 1.27)	0.845
College graduate			Referent	–
**Marital status**				
Married	2.16 (1.28, 3.63)	0.004	–	–
Separated, divorced, or widowed	1.59 (0.94, 2.70)	0.086		
Never married	Referent	–		
**Smoking status**	–	–		
≥ 100 lifetime cigarettes			1.30 (1.03, 1.63)	0.025
< 100 lifetime cigarettes			Referent	–

^a^Odds ratio

^b^Confidence interval

Race and smoking status had significant associations with pre-diabetes status within cluster counties, but not the rest of the state (outside cluster counties). Compared to being white, being non-Hispanic black was associated with higher odds of pre-diabetes (OR = 2.40, 95% CI: 1.19, 4.87). Age, however, was a significant predictor of pre-diabetes status only among residents of non-cluster counties. Being 55–64 years (OR = 1.43, 95% CI: 1.06, 1.94) or ≥ 65 years of age (OR = 1.67, 95% CI: 1.28, 2.18) were associated with significantly higher odds of pre-diabetes in comparison to being 18–54 years of age.

**Fig. 4:**
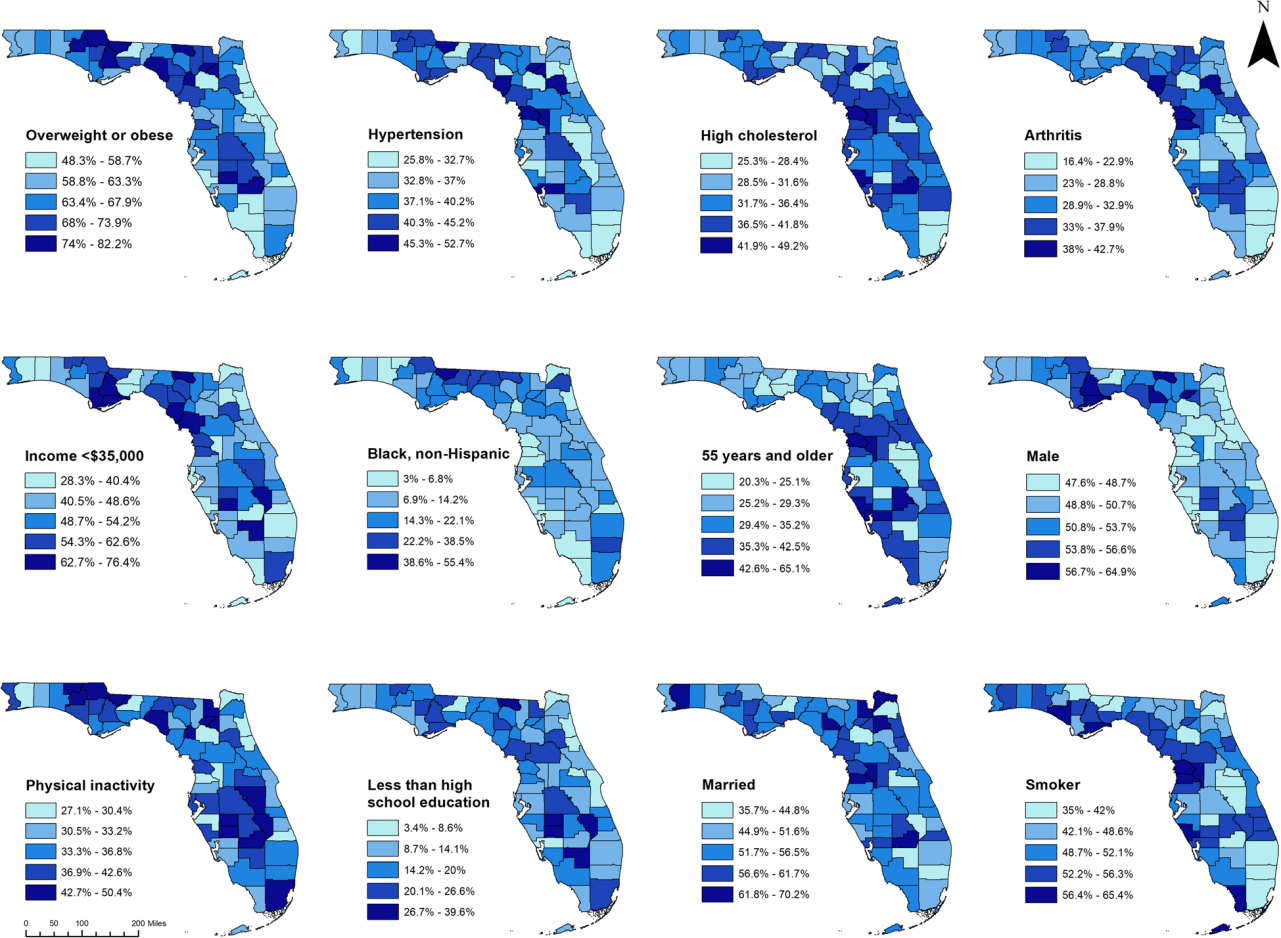
Significant predictors of pre-diabetes and/or diabetes, Florida, 2013. The map was created by the authors of the study using ArcGIS software [18]. The geographic boundary file was obtained from the United States Census Bureau TIGER Geodatabase [19]. These files are publicly available, and permission is not required to reproduce the image

The following variables had statistically significant associations with diabetes status regardless of location: BMI, hypertension, hypercholesterolemia, income, age, and physical activity level. As with the pre-diabetes models, the strength of some of the associations seemed to be higher in non-cluster counties than in cluster counties, however, these differences were not statistically significant. For instance, among non-cluster counties, the strength of association between diabetes and obesity (OR_obesity_ = 3.68, 95% CI: 2.84, 4.76) and overweight (OR_overweight_ = 2.06, 95% CI: 1.56, 2.73) seemed higher had than those in cluster counties (OR_obesity_ = 2.99, 95% CI: 2.04, 4.40; OR_overweight_ = 1.62, 95% CI: 1.09, 2.43) but the ORs were not significantly different on account of their overlapping CIs. Income, on the other hand, had a seemingly stronger association with diabetes within high-prevalence clusters than in non-cluster counties but again, the ORs were not significantly different. Suffice it to say that in areas that were part of the high-prevalence cluster having an income of <$15,000 was associated with more than 3 times higher odds of diabetes (OR = 3.34, 95% CI: 2.11, 5.27) compared to having an annual income of ≥$50,000. However, for respondents that were not part of a high-prevalence diabetes cluster, having an income of <$15,000 was associated with 1.6 times higher odds of diabetes (OR = 1.63, 95% CI: 1.10, 2.42) compared to having an annual income of ≥$50,000. Arthritis, sex, and marital status had statistically significant associations with diabetes only among residents of cluster counties, while educational attainment and smoking status were only associated with diabetes among residents of non-cluster counties (Table 4). No biologically meaningful significant interactions were detected. The geographic distributions of the identified significant predictors of pre-diabetes and diabetes are displayed in maps in Fig. 4.

## Discussion

This study investigated county-level geographic disparities and predictors of pre-diabetes and diabetes prevalence reported in the 2013 Florida Behavioral Risk Factor Surveillance System (BRFSS) data. Study findings provide information that is useful for guiding resource allocation for prevention and control programs.

### Spatial patterns and clusters

The results of this study indicate that spatial patterns of high prevalence of pre-diabetes and diabetes exist at the county level in Florida. This is consistent with findings from other studies which have reported that counties within the southeastern United States, including Florida, are located in the diabetes belt and have excess risk of diabetes [17, 23]. The high prevalence in these areas may be attributed, in part, to differences in the distribution of known risk factors, including obesity/overweight, dietary composition, socioeconomic status (SES), and comorbidities such as hypertension and hypercholesterolemia [24–26]. However, other studies have reported the persistence of geographic disparities even after adjusting for variations in these known risk factors [23, 27]. For both conditions, understanding geographic disparities is critical for helping public health officials identify priority areas for intervention, so that areas at high risk may be targeted for implementation of health programs aimed at reducing disease burden.

The major strengths of the spatial scan statistics used to detect and identify high-prevalence clusters are: (a) it does not have the problem of multiple comparisons, and (b) it identifies clusters without prior specification of their suspected location or size, thus eliminating pre-selection bias. Interestingly, while there was much overlap between counties within high-prevalence pre-diabetes and diabetes clusters, the distribution of the conditions exhibited somewhat differing spatial patterns. The primary pre-diabetes cluster was large and relatively diverse in terms of urbanization, containing both metropolitan and rural counties, and encompassing the entirety of the secondary diabetes cluster in this region. The primary diabetes cluster, which was composed mainly of rural counties, overlapped with the two counties that comprised a secondary pre-diabetes cluster. A national study found a higher prevalence of diabetes in rural areas compared to urban areas, and that rural residents were less likely to receive diabetes education [28]. Inadequate or lack of public transportation and long distance to healthcare facilities in rural areas may limit access to healthcare services [29, 30]. Based on Health Resources and Services Administration (HRSA) criteria, three of the eight counties in the primary diabetes cluster, as well as the secondary cluster adjacent to it (Hendry County), are considered geographic Health Professional Shortage Areas (HPSAs), implying that these areas have a shortage of primary care providers [31]. Cluster 4, located in the eastern panhandle, is also mainly comprised of rural counties. Additionally, four of the eight counties within cluster 2 are geographic HPSAs [31].

In addition to influencing accessibility of healthcare services, geographic characteristics can affect health through their impact on food access. Dietary intake is an important part of managing and preventing diabetes, and eating patterns may be influenced by access to healthy food options.

### Multivariable logistic regression

Consistent with findings from other studies [8, 32–37], the results of multivariable logistic regression models from this study showed that hypertension, hypercholesterolemia and overweight/obesity are predictors of both pre-diabetes and diabetes. Previous studies identified geographic and racial disparities in the distribution of these risk factors for diabetes [23, 27]. In addition, results from the current study showed higher odds of diabetes with increasing age, consistent with findings from other studies [34, 35, 37–41]. While increasing age was also a predictor of pre-diabetes status among those living outside of cluster counties, a significant association was not observed within cluster counties.

Within high-prevalence clusters, sex was significantly associated with diabetes, with males having higher odds of diabetes than females. Globally, diabetes prevalence is higher in males (9.1%) than females (8.4%), with an estimated 17.1 million more males having diabetes than females. However, sex differences in diabetes prevalence vary by country and age group [42]. This trend is also seen in the United States, where the overall prevalence of diabetes is slightly higher among men (9.4%) than women (9.2%), with sex differences varying depending on race/ethnicity [10]. Studies of diabetes patients in Europe and the U.S. have shown an inverse relationship between BMI and age in those diagnosed with type 2 diabetes, and that this relationship is affected by sex; at the time of diabetes diagnosis, BMI tends to be lower in men than women [43, 44]. A comprehensive review on gender and diabetes identified three major contributors to varying insulin sensitivity between men and women: the activity of estrogen, and differences in adipose tissue distribution and adipokine secretion [45].

In the current study, race was significantly associated with pre-diabetes, but this association was only observed among residents of high-prevalence clusters. Similar to these findings, the Reasons for Geographic and Racial Differences in Stroke (REGARDS) study found that pre-diabetes was more commonly reported among black participants in comparison to white participants [2]. However, in contrast to our findings, the association between race and pre-diabetes among REGARDS participants persisted regardless of region [2].

Although race was associated with pre-diabetes among residents of high-prediabetes clusters in this study, there was no association between race and diabetes status. However, disparities across racial and ethnic groups in terms of diabetes prevalence, quality of care, and health outcomes, are among the widespread health inequalities consistently identified in the United States described in the Institute of Medicine report *Unequal Treatment* [46]. According to CDC estimates, age-adjusted prevalence of diabetes is lowest among non-Hispanic white and Asian populations, and highest among American Indian/Alaska Native and non-Hispanic black populations [10]. Some studies have suggested that observed racial and ethnic diabetes disparities may be a reflection of differences in socioeconomic factors that are associated with race [47, 48]. For example, the Boston Area Community Health (BACH) survey reported SES as a stronger relative predictor of diabetes risk than race/ethnicity, and found race/ethnicity to be non-significant after the inclusion of additional socioeconomic indicators in a regression model [47]. Another study, using data from the National Health and Nutrition Examination Survey (NHANES), identified interactions between race/ethnicity and other predictors of diabetes risk, namely individual and neighborhood poverty [49]. Regardless of area of residence (high-prevalence cluster areas or non-cluster areas), respondents that were physically inactive had significantly higher odds of diabetes compared to those who were highly active. Interestingly, among those that lived outside high-prevalence diabetes clusters, there were no differences in the odds of diabetes between those that were highly active and those that were either insufficiently active or active implying that any level of physical activity among those living outside high-prevalence diabetes cluster might be beneficial. By contrast, among those in high-prevalence diabetes clusters, both being inactive and being insufficiently active had higher odds of diabetes compared to being highly active. However, there was no difference in the odds of diabetes between being highly active and being active among those living in high prevalence clusters. This is an interesting finding that warrants further investigations.

Some studies have shown a gradient of decreased diabetes risk with increasing activity level. For example, a prospective study of Finnish men and women showed a protective effect of increasing physical activity level, a trend that was observed in individuals with both obese and normal BMI [50]. The American Diabetes Association (ADA) recommends that people with diabetes engage in at least 150 min of moderate-to-vigorous intensity exercise per week, ideally exercising daily and incorporating both aerobic and resistance training [51]. The fact that insufficient physical activity level was associated with higher odds of diabetes within cluster counties highlights the importance of following recommended physical activity guidelines. A growing body of research suggests that physical inactivity itself has detrimental effects on metabolism leading to increased diabetes risk including: insulin resistance, dyslipidemia, and impaired glucose tolerance [52, 53]. Indeed, a U.K. study found higher levels of fasting insulin to be associated with time spent sedentary in a cohort of healthy, middle-aged Caucasian participants, independent of moderate- and vigorous-intensity exercise [54]. Moreover, changing the time spent being sedentary to either light or moderate-to-vigorous intensity physical activity has been shown to increase insulin sensitivity in people with impaired glucose regulation [55]. Thus, the recommendations of the ADA include reducing time spent in sedentary behaviors, and performing light activity at least every 30 min during prolonged sitting [51].

Among residents of cluster counties, increased odds of diabetes were significantly associated with arthritis, a chronic condition with higher prevalence in older age groups [56]. Arthritis has been previously identified as a comorbidity in diabetic patients [57]. Physical inactivity has been associated with arthritis and diabetes in older adults, suggesting that arthritic pain presents a challenge for lifestyle interventions used to prevent and manage diabetes [58, 59]. The association between arthritis and diabetes did not persist outside of high-prevalence clusters, highlighting the importance of identifying areas with populations at high risk, and investigating predictors of the condition within those populations. Such information is useful in order to inform evidence-based health programming. These findings suggest that residents of high-prevalence clusters, in particular, may benefit from intervention programs which take comorbidities such as arthritis into account, and consider this potential barrier to adhering to recommended physical activity guidelines.

The current study also found a significant association between income level and diabetes. Outside of high-prevalence clusters, educational attainment was also significantly associated with diabetes status. These associations are consistent with evidence in the literature that has established associations between diabetes prevalence and indicators of SES [47, 60–62]. Economic stability and education are two of the social determinants of health established by Healthy People 2020, and are related to reported proximal risk factors for diabetes, including tobacco use, alcohol consumption, and poor dietary quality [9, 63–68]. Interestingly, consumption of fruits or vegetables were not associated with either diabetes or pre-diabetes in the final model. Cigarette smoking was a significant predictor of diabetes status for residents of non-cluster counties. In contrast, it was significantly associated with pre-diabetes status only within high-prevalence clusters. The observed differences in predictors of the conditions based on residence within versus outside of cluster counties suggests that the importance of risk factors varies by region, and these may reflect population characteristics. Tailored public health programming based upon these findings may be used to more effectively target those at high risk.

### Strengths and limitations

To our knowledge, this is the first study to explore spatial patterns and clusters of pre-diabetes and diabetes risk in Florida using rigorous spatial epidemiologic methods. Findings from studies using these approaches are useful for health planning purposes to target high-prevalence counties where preventive resources are most needed. This study also demonstrated the usefulness of spatial statistics cluster detection methods, and GIS in identifying areas at highest risk of pre-diabetes and diabetes. Key strengths of the spatial scan statistics used to detect and identify high-prevalence clusters are that it does not have the problem of multiple comparisons and it identifies clusters without prior specification of their suspected location or size, thus eliminating pre-selection bias.

However, this study is not without limitations. The cross-sectional nature of BRFSS limits the ability to draw causal inference. Additionally, use of survey data has inherent problems of potential bias associated with self-reporting for variables such as physical activity and BMI. Self-reports of physical activity have been shown to be influenced by factors such as questionnaire format, gender, and BMI [69, 70]. BRFSS data also do not distinguish between type 1 and type 2 diabetes, two conditions with different pathogenesis and risk factors [42]. However, type 2 diabetes accounts for the vast majority of diabetes, an estimated 90–95% of the cases in the United States [71]. In addition, the majority of pre-diabetes cases are undiagnosed, and those reported here likely underrepresent the true burden of the condition in the population [10, 72]. Lastly, the logistic regression models built in this study focused on individual-level predictors of diabetes and pre-diabetes status, and did not investigate county-level determinants such as county-level socioeconomic conditions, density of fast-food restaurants and the built environment. Future studies will need to investigate these factors. These limitations, notwithstanding, the findings for this study are important in guiding prevention and control programs.

## Conclusions

This study identified spatial clusters of high pre-diabetes and diabetes prevalence at the county level, indicating existence of geographic disparities. It also identified significant predictors of the two conditions and that the importance of the predictors differed between high-prevalence cluster counties and the rest of the state. The study also demonstrated the usefulness of spatial statistics cluster detection methods and GIS in identifying areas at highest risk of pre-diabetes and diabetes. This is useful for guiding resource allocation for prevention and control programs.

## Supplementary information

**Additional file 1.** Demographic, health, and lifestyle characteristics of adults living within and outside of high-prevalence pre-diabetes clusters in Florida, 2013.

**Additional file 2.** Demographic, health, and lifestyle characteristics of adults living inside and outside of high-prevalence diabetes clusters in Florida, 2013.

## Data Availability

This data belongs to a third party (Florida Department of Health). The authors do not have the legal authority to share the data. However, it can be requested from the Florida Department of Health by contacting the Director of the Division of Community Health Promotion by mail at 4052 Bald Cypress Way, Mail Bin A13 Tallahassee, FL 32399 or by phone at 850–245-4391, or by email at CommunityHealthPromotion@flhealth.gov.

## Abbreviations

BRFSS: Behavioral Risk Factor Surveillance System
SEB: Spatial Empirical Bayes
FPG: Fasting plasma glucose
CDC: Centers for Disease Control and Prevention
BMI: Body mass index
NCHS: National Center for Health Statistics
GIS: Geographic information system
RR: Relative risk
CI: Confidence interval
OR: Odds ratio
HPSA: Health Professional Shortage Area
HRSA: Health Resources and Services Administration
USDA: United States Department of Agriculture
NHANES: National Health and Nutrition Examination Survey
BACH: Boston Area Community Health
SES: Socioeconomic status
ADA: American Diabetes Association
