# Supplementary material for: Investigation of geographic disparities of pre-diabetes and diabetes in Florida
Source: BMC Public Health. 2020 Aug 12;20:1226. doi: 10.1186/s12889-020-09311-2 (PMC7425001; doi:10.1186/s12889-020-09311-2)
Supplement: Supplementary file 1 — Additional file 1. Demographic, health, and lifestyle characteristics of adults living within and outside of high-prevalence pre-diabetes clusters in Florida, 2013. [file 12889_2020_9311_MOESM1_ESM.docx]

**Appendix 1: Demographic, health, and lifestyle characteristics of adults living within and outside of high-prevalence pre-diabetes clusters in Florida, 2013**

| **Characteristic** | **Non-cluster counties** | | | **Cluster counties** | | |
| --- | --- | --- | --- | --- | --- | --- |
|  | **Unweighted Frequency** | **Weighted frequency** | **Weighted %**  **(95% Confidence Interval)** | **Unweighted Frequency** | **Weighted frequency** | **Weighted %**  **(95% Confidence Interval)** |
| **Diabetes status**  Diabetes  No diabetes | *n* = 26,245  3,922  22,323 | 1,419,483  11,708,454 | 10.81 (10.00, 11.63)  89.19 (89.37, 90.00) | *n* = 7,863  1,267  6,596 | 317,425  2,104,107 | 13.11 (11.98, 14.23)  86.89 (85.77, 88.02) |
| **Pre-diabetes status**  Pre-diabetes  No pre-diabetes | *n* = 21,773  2,243  19,530 | 836,707  10,124,820 | 7.63 (6.93, 8.33)  92.37 (91.67, 93.07) | *n* = 6,466  740  5,726 | 215,377  1,804,691 | 10.66 (9.32, 12.00)  89.33 (88.00, 90.68) |
| **BMI (kg/m^2^)**  Underweight (< 18.5)  Normal (18.5-24.9)  Overweight (25–29.9)  Obese (≥ 30) | *n* = 25,050  555  8,317  8.953  7,225 | 286,321  4,398,171  4,576,047  3,209,244 | 2.30 (1.90, 2.69)  35.27 (33.92, 36.62)  36.70 (35.32, 38.08)  25.74 (24.53, 26.94) | *n* = 7,502  165  2,498  2,644  2,195 | 48,814  764,468  798,916  685,904 | 2.12 (1.49, 2.75)  33.27 (31.39, 35.14)  34.76 (32.85, 36.68)  29.85 (28.02, 31.68) |
| **Hypertension**  Yes  No | *n* = 26,214  11,918  14,296 | 4,425,933  8,690,615 | 33.74 (32.49, 34.99)  66.26 (65.01, 67.51) | *n* = 7,860  3,766  4,094 | 949,010  1,475,248 | 39.15 (37.33, 40.96)  60.85 (59.04, 62.67) |
| **Hypercholesterolemia**  Yes  No | *n* = 23,115  10,994  12,121 | 4,213,967  6,390,624 | 39.74 (38.34, 41.14)  60.26 (58.86, 61.66) | *n* = 7,101  3,451  3,650 | 873,147  1,134,521 | 43.49 (41.51, 45.47)  56.51 (54.53, 58.49) |
| **Arthriti**s  Yes  No | *n* = 26,075  9,978  16,097 | 3,266,362  9,779,242 | 25.04 (24.00, 26.08)  74.96 (73.92, 76.00) | *n* = 7,822  3,264  4,558 | 755,140  1,657,396 | 31.30 (29.66, 32.94)  68.70 (67.06, 70.34) |
| **Income level**  < $15,000  $15,000 - < $25,000  $25,000 - < $35,000  $35,000 - < $50,000  > $50,000 | *n* = 22,502  3,290  4,864  2,860  3,334  8.154 | 1,677,070  2,298,131  1,384,532  1,639,542  4,462,498 | 14.63 (13.49, 15.78)  20.05 (18.83, 21.27)  12.08 (11.09, 13.07)  14.30 (13.30, 15.31)  38.93 (37.54, 40.33) | *n* = 6,669  932  1,526  938  1,081  2,192 | 276,100  467,624  302,240  355,414  696,126 | 13.16 (11.63, 14.70)  22.29 (20.47, 24.11)  14.41 (12.99, 15.83)  16.94 (15.32, 18.57)  33.19 (31.25, 35.13) |
| **Health care coverage**  Yes  No | *n* = 26,167  22,374  3,793 | 10,024,121  3,051,432 | 76.66 (75.39, 77.93)  23.34 (22.07, 24.61) | *n* = 7,836  6,771  1,065 | 1,919,590  488,813 | 79.70 (77.96, 81.45)  20.30 (18.55, 22.04) |
| **Race**  White non-Hispanic  Black non-Hispanic  Other race non-Hispanic  Hispanic | *n* = 26,306  20,747  2,466  1,071  2,022 | 7,469,236  1,913,578  585,651  3,189,896 | 56.76 (55.41, 58.12)  14.54 (13.46, 15.62)  4.45 (3.92, 4.98)  24.24 (22.83, 25.65) | *n* = 7,880  6,621  481  282  496 | 1,822,263  257,216  85,426  264,356 | 75.01 (73.07, 76.96)  10.59 (9.07, 12.10)  3.52, 2.87, 4.16)  10.88 (9.38, 12.38) |
| **Age, years**  18-24  25-34  35- 44  45-54  55-64  65 or older | *n* = 26,306  1,123  2,060  2,610  4,060  5,616  10,837 | 1,542,488  2,062,755  2,082,652  2,351,385  2,147,346  2,971,735 | 11.72 (10.66, 12.78)  15.68 (14.59, 16.76)  15.83 (14.78, 16.89)  17.87 (16.80, 18.94)  16.32 (15.37, 17.27)  22.58 (21.70, 23.46) | *n* = 7,880  254  458  586  1,089  1,715  3,778 | 255,591  349,745  314,701  390,029  426,643  692,552 | 10.52 (8.96, 12.08)  14.40 (12.69, 16.10)  12.95 (11.48, 14.43)  16.06 (14.69, 17.42)  17.56 (16.28, 18.85)  28.51 (27.16, 29.85) |
| **Sex**  Male  Female | *n* = 26,306  10,295  16,011 | 6,362,829  6,795,531 | 48.36 (46.98, 49.74)  51.64 (50.25, 53.02) | *n* = 7,880  3,045  4,835 | 1,175,892  1,253,369 | 48.41 (46.45, 50.36)  51.59 (49.64, 53.55) |
| **Physical activity**  Highly Active^a^  Active^b^  Insufficiently Active^c^  Inactive | *n* = 21,574  7,286  3,223  3,287  7,778 | 3,081,062  1,584,437  1,663,050  3,651,410 | 30.87 (29.48, 32.26)  15.88 (14.73, 17.02)  16.66 (15.51, 17.82)  36.59 (35.05, 38.12) | *n* = 6,623  2,402  929  958  2,334 | 649,196  283,924  305,142  670,736 | 34.01 (32.05, 35.96)  14.87 (13.37, 16.37)  15.98 (14.30, 17.67)  35.14 (33.08, 37.19) |
| **Education**  < High school  High school  Some college  College | *n* = 26,182  2,591  8,100  7,529  7,962 | 1,950,068  3,840,038  4,065,718  3,239,536 | 14.89 (13.64, 16.14)  29.32 (28.03, 30.62)  31.05 (29.81, 32.28)  24.74 (23.72, 25.76) | *n* = 7,832  789  2,530  2,324  2,189 | 372,661  836,782  769,730  437,606 | 15.42 (13.66, 17.18)  34.62 (32.74, 36.50)  31.85 (30.04, 33.66)  18.11 (16.96, 19.25) |
| **Marital status**  Married  Never married  Separated/divorced/ widowed | *n* = 26,105  12,800  3,758  9.547 | 6,398,065  3,555,072  3,090,874 | 49.05 (47.68, 50.42)  27.25 (25.93, 28.57)  23.70 (22.58, 24.81) | *n* = 7,812  4,032  928  2,852 | 1,338,812  514,978  559,909 | 55.47 (53.51, 57.42)  21.34 (19.48, 23.19)  23.20 (21.73, 24.66) |
| **Consume vegetable(s)**  < 1 per day  > 1 per day | *n* = 23,295  4,568  18,727 | 2,487,800  9,359,243 | 21.00 (19.80, 22.20)  79.00 (77.80, 80.20) | *n* = 7,020  1,256  5,764 | 432,833  1,739,526 | 19.92 (18.21, 21.64)  80.08 (78.36, 81.79) |
| **Consume fruit(s)**  < 1 per day  > 1 per day | *n* = 23,822  8,939  14,883 | 4,496,758  7,499,397 | 37.49 (36.09, 38.88)  62.51 (61.12, 63.91) | *n* = 7,156  2,649  4,507 | 895,853  1,319,287 | 40.44 (38.41, 42.47)  59.56 (57.53, 61.59) |
| **Smoked > 100 cigarettes**  Yes  No | *n* = 25,427  12,676  12,751 | 5,550,910  7,063,928 | 44.00 (42.64, 45.36)  55.00 (54.64, 57.36) | *n* = 7,651  4,003  3,648 | 1,193,502  1,155,841 | 50.80 (48.81, 52.79)  49.20 (47.21, 51.19) |

^a^Highly Active: ≥300 min of moderately intense or vigorous equivalent per week

^b^Active: 150-300 min of moderately intense or vigorous equiv./week

^c^Insufficiently Active: 1-149min of moderately intense exercise/week
