# Supplementary material for: Investigation of geographic disparities of pre-diabetes and diabetes in Florida
Source: BMC Public Health. 2020 Aug 12;20:1226. doi: 10.1186/s12889-020-09311-2 (PMC7425001; doi:10.1186/s12889-020-09311-2)
Supplement: Supplementary file 2 — Additional file 2. Demographic, health, and lifestyle characteristics of adults living inside and outside of high-prevalence diabetes clusters in Florida, 2013. [file 12889_2020_9311_MOESM2_ESM.docx]

**Appendix 2: Demographic, health, and lifestyle characteristics of adults living inside and outside of high-prevalence diabetes clusters in Florida, 2013**

| **Characteristic** | **Non-cluster counties** | | | **Cluster counties** | | |
| --- | --- | --- | --- | --- | --- | --- |
|  | **Unweighted Frequency** | **Weighted frequency** | **Weighted %**  **(95% Confidence Interval)** | **Unweighted Frequency** | **Weighted frequency** | **Weighted %**  **(95% Confidence Interval)** |
| **Diabetes status**  Diabetes  No diabetes | *n* = 23,844  3,270  20,574 | 1,418,088  11,998,963 | 10.57 (9.77, 11.37)  89.43 (88.63, 90.23) | *n* = 10,264  1,919  8,345 | 318,820  1,813,599 | 14.95 (13.71, 16.19)  85.04 (83.81, 86.29) |
| **Pre-diabetes status**  Pre-diabetes  No pre-diabetes | *n =* 20,006  1,984  18,022 | 876,993  10,343,369 | 7.82 (7.12, 8.51)  92.18 (91.49, 92.88) | *n* = 8,233  999  7,234 | 175,091  1,586,142 | 9.94 (8.63, 11.25)  90.06 (88.75, 91.37) |
| **BMI (kg/m^2^)**  Underweight (< 18.5)  Normal (18.5-24.9)  Overweight (25–29.9)  Obese (≥ 30) | *n* = 22,767  519  7,906  8,098  6,244 | 300,613  4,530,871  4,648,517  3,249,413 | 2.36 (1.97, 2.76)  35.59 (34.26, 36.93)  36.52 (35.16, 37.88)  25.53 (24.34, 26.71) | *n* = 9,785  201  2,909  3,499  3,176 | 34,522  631,769  726,446  645,735 | 1.69 (1.23, 2.15)  30.99 (29.03, 32.95)  35.64 (33.63, 37.64)  31.68 (29.73, 33.62) |
| **Hypertension**  Yes  No | *n* = 23,812  10,532  13,280 | 4,516,443  8,889,758 | 33.69 (32.46, 34.92)  66.31 (65.08, 67.54) | *n* = 10,262  5,152  5,110 | 858,500  1,276,105 | 40.22 (38.30, 42.14)  59.78 (57.86, 61.70) |
| **Hypercholesterolemia**  Yes  No | *n* = 20,983  9,779  11,204 | 4,286,239  6,561,140 | 39.51 (38.14, 40.89)  60.49 (59.11, 61.86) | *n* = 9,233  4,666  4,567 | 800,875  964,006 | 45.38 (43.29, 47.46)  54.62 (52.54, 56.71) |
| **Arthriti**s  Yes  No | *n* =23,691  8.870  14,821 | 3,345,136  9,984,720 | 25.10 (24.07, 26.12)  74.90 (73.88, 75.93) | *n* = 10,206  4,372  5,834 | 676,366  1,451,918 | 31.78 (30.08, 33.48)  68.22 (66.52, 69.92) |
| **Income level**  < $15,000  $15,000 - < $25,000  $25,000 - < $35,000  $35,000 - < $50,000  > $50,000 | *n* = 20,500  2,762  4,192  2,581  3,031  7,934 | 1,673,460  2,315,132  1,432,746  1,693,175  4,616,930 | 14.26 (13.14, 15.39)  19.73 (18.54, 20.93)  12.21 (11.24, 13.19)  14.43 (13.44, 15.43)  39.36 (37.98, 40.73) | *n* = 8,671  1,460  2,198  1,217  1,384  2,412 | 279,711  450,623  254,026  301,781  541,694 | 15.30 (13.55, 17.05)  24.65 (22.69, 26.61)  13.90 (12.46, 15.34)  16.51 (14.81, 18.21)  29.64 (27.68, 31.59) |
| **Health care coverage**  Yes  No | *n* = 23,765  20,369  3,396 | 10,296,381  3,063,665 | 77.07 (75.82, 78.32)  22.93 (21.68, 24.18) | *n* = 10,238  8,776  1,462 | 1,647,331  476,580 | 77.56 (75.64, 79.48)  22.44 (20.52, 24.36) |
| **Race**  White non-Hispanic  Black non-Hispanic  Other race non-Hispanic  Hispanic | *n* = 23,888  19,004  2,061  1,034  1,789 | 7,779,042  1,924,801  609,727  3,131,576 | 57.86 (56.52, 59.20)  14.32 (13.26, 15.38)  4.53 (4.01, 5.06)  23.29 (21.91, 24.68) | *n* =10,298  8,364  886  319  729 | 1,512,457  245,993  61,350  322,676 | 70.59 (68.48, 72.71)  11.48 (9.85, 13.11)  2.86 (2.27, 3.46)  15.06 (13.28, 16.84) |
| **Age, years**  18-24  25-34  35- 44  45-54  55-64  65 or older | *n* = 23,888  1,103  1,914  2,367  3,686  5,078  9,740 | 1,600,391  2,101,402  2,112,896  2,398,270  2,208,993  3,023,194 | 11.90 (10.86, 12.95)  15.63 (14.56, 16.70)  15.71 (14.67, 16.76)  17.84 (16.79, 18.88)  16.43 (15.49, 17.37)  22.49 (21.62, 23.35) | *n* = 10,298  274  604  829  1,463  2,253  4,875 | 197,688  311,098  284,457  343,144  364,996  641,093 | 9.23 (7.66, 10.79)  14.52 (12.71, 16.33)  13.28 (11.72, 14.84)  16.02 (14.55, 17.48)  17.04 (15.71, 18.36)  29.92 (28.48, 31.37) |
| **Sex**  Male  Female | *n* = 23,888  9,549  14,339 | 6,492,089  6,953,057 | 48.29 (46.93, 49.64)  51.71 (50.36, 53.07) | *n* = 10,298  3,791  6,507 | 1,046,633  1,095,843 | 48.85 (46.81, 50.89)  51.15 (49.11, 53.19) |
| **Physical activity**  Highly Active^a^  Active^b^  Insufficiently Active^c^  Inactive | *n* = 19,462  6,786  2,948  2,992  6,736 | 3,187,699  1,606,714  1,700,400  3,683,661 | 31.32 (29.95, 32.69)  15.79 (14.66, 16.91)  16.71 (15.57, 17.84)  36.19 (34.68, 37.70) | *n* = 8,735  2,902  1,204  1,253  3,376 | 542,559  261,646  267,791  638,484 | 31.72 (29.74, 33.70)  15.30 (13.70, 16.90)  15.66 (13.90, 17.41)  37.33 (35.15, 39.50) |
| **Education**  < High school  High school  Some college  College | *n* = 23,763  2,048  6,788  6.987  7.940 | 1,937,727  3,862,435  4,212,979  3,368,133 | 14.48 (13.25, 15.71)  28.86 (27.59, 30.14)  31.48 (30.26, 32.70)  25.17 (24.16, 26.18) | *n* = 10,251  1,332  3,842  2,866  2,211 | 385,002  814,384  622,469  309,010 | 18.07 (16.16, 19.98)  38.22 (36.22, 40.22)  29.21 (27.41, 31.02)  14.50 (13.44, 15.56) |
| **Marital status**  Married  Never married  Separated/divorced/ widowed | *n* = 23,688  11,630  3,591  8,467 | 6,515,744  3,651,622  3,158,105 | 48.90 (47.55, 50.24)  27.40 (26.11, 28.70)  23.70 (22.61, 24.79) | *n* = 10,229  5,202  1,095  3,932 | 1,221,133  418,428  492,677 | 57.27 (55.23, 59.31)  19.62 (17.70, 21.55)  23.11 (21.61, 24.61) |
| **Consume vegetable(s)**  < 1 per day  > 1 per day | *n* = 21,299  4,021  17,278 | 2,500,851  9,620,787 | 20.63 (19.46, 21.81)  79.37 (78.19, 80.54) | *n* = 9,016  1,803  7,213 | 419,782  1,477,982 | 22.12 (20.24, 24.00)  77.88 (76.00, 79.76) |
| **Consume fruit(s)**  < 1 per day  > 1 per day | *n* = 21,724  7.913  13,811 | 4,584,779  7,691,435 | 37.35 (35.97, 38.72)  62.65 (61.28, 64.03) | *n* = 9,254  3,675  5,579 | 807,833  1,127,248 | 41.75 (39.61, 43.89)  58.25 (56.11, 60.39) |
| **Smoked > 100 cigarettes**  Yes  No | *n* = 23,115  11,549  11,566 | 5,697,112  7,199,579 | 44.17 (42.84, 45.51)  55.83 (54.49, 57.17) | *n* = 9,963  5,130  4,833 | 1,047,300  1,020,172 | 50.66 (48.57, 52.74)  49.34 (47.26, 51.43) |

^a^Highly Active: ≥300 min of moderately intense or vigorous equivalent per week

^b^Active: 150-300 min of moderately intense or vigorous equiv./week

^c^Insufficiently Active: 1-149min of moderately intense exercise/week
